# Cultural adaptation and validation of the Children's Eating Behaviour Questionnaire in Ethiopia

**DOI:** 10.1017/jns.2021.45

**Published:** 2021-07-21

**Authors:** Nardos Wondafrash Gebru, Seifu Hagos Gebreyesus, Hamid Yimam Hassen, Esete Habtemariam, Dawit Shawel Abebe

**Affiliations:** 1 Department of Nutrition and Dietetics, School of Public Health, Addis Ababa University, 9086 Addis Ababa, Ethiopia; 2 Department of Primary and Interdisciplinary Care, Faculty of Medicine and Health Sciences, University of Antwerp, Antwerp, Belgium; 3 Department of Nursing and Health Promotion, Oslo Metropolitan University, Postboks 4, St. Olavs plass, 0130 Oslo, Norway

**Keywords:** BMI, CEBQ, Children, Culture, Ethiopia, Validation

## Abstract

Eating behaviours have been associated both with being underweight or overweight and poor growth. The Children's Eating Behaviour Questionnaire (CEBQ) is a widely used measure of child eating behaviours. The instrument is, however, mostly validated in high-income countries, with a scarcity of evidence among developing countries such as Ethiopia. The present study aims to assess the cultural adaptability and validity of the CEBQ to be used in Ethiopia. We conducted a school-based cross-sectional study among 542 caregivers of children aged 3–6 years in selected preschools. Tests of factorial validity, convergent validity and reliability were performed. The Confirmatory Factor Analysis model indicated that eight subscales provided the best fit (root-mean-square error of approximation = 0⋅05 (90 % CI 0⋅045, 0⋅055); Comparative Fit Index = 0⋅92 and Tucker–Lewis Index = 0⋅90) after seven items from the original CEBQ were removed. Convergent validity with child's weight status was found for emotional overeating, food fussiness, satiety responsiveness and slowness in eating subscales. Reliability, measured using Cronbach's *α*, provided values between 0⋅50 and 0⋅79. The eight-factor structure of the CEBQ showed adequate content validity and provided factorial, discriminant and convergent validity among preschool children. Further replication of the study among low-income countries is essential to improve the literature on children's eating behaviours.

## Introduction

Eating behaviours are physical and behavioural processes mainly focused on meeting the requirements for health and growth, and they evolve during the first years of life^(1)^. Eating behaviours may vary on a range from picky eating to overeating or binge eating^(2,3)^. A wide range of prevalence rates have been reported due to variability in definition; it is estimated that approximately 25 % of normally developing children, and up to 80 % of children with developmental delays, display some type of feeding problems^(4,5)^.

Measurement methods that are feasible and sensitive to cultural differences are essential for understanding eating behaviour differences. Although direct observation may be considered the ideal method, it is extremely costly and has been found to interfere with the typical behaviours of the participant, particularly those under investigation^(6)^. In response to methodological difficulties in studying eating behaviours, some standardised feasible psychometric tools have been developed to assess children's eating behaviour, namely the Dutch Eating Behaviour Questionnaire (DEBQ)^(7)^, the Children's Eating Behaviour Inventory (CEBI)^(8)^ and the Children's Eating Behaviour Questionnaire (CEBQ)^(9)^. The latter is the most comprehensive and widely used parent-report tool that assesses children's eating styles in terms of both obesity risk and undereating. The tool has been found to have a high reliability and validity in several countries^(10–12)^. The original CEBQ is founded on an 8-scale conceptualisation of eating styles which can be categorised as food approach and food avoidance. The food approach category consists of (1) the desire to drink scale, which was developed to detect an increased desire for drink, particularly sugar-sweetened drinks^(9)^, (2) the enjoyment of food scale which represents a general interest in food and (3) the food responsiveness scale that measures eating in response to external food cues^(9,13)^. The food avoidance category consists of (1) the satiety responsiveness scale, which reflects the ability to regulate the amount of food that is eaten according to internal satiety cues^(12)^, and (2) the slowness in eating scale which measures the speed of eating during the course of a meal. A reduction in speed reflects a loss of interest in a meal^(12)^ while (3) food fussiness reflects a lack of interest in food or being selective of certain types of food and an unwillingness to try new foods (food neophobia) leading to the consumption an inadequate variety of foods^(9,14)^. Finally, the scales of emotional overeating and emotional undereating are characterised by either increased or decreased eating in response to negative emotions such as anger and anxiety^(9)^.

Several studies have assessed the applicability of the CEBQ in populations other than the one in which it was developed (i.e. well-educated parents of children aged 2–9 years living in the UK). Most of the studies, however, were conducted among wealthier countries such as Sweden^(13)^, the Netherlands^(12)^, the USA^(10)^ and China^(15)^. Problems at both the item and factor level have emerged in these studies. For example, the validation study conducted in China indicated that the CEBQ in its original form may not be culturally appropriate for non-Western populations and proposed an alternative 19-item version of the tool (based on Principal Component Analysis (PCA))^(15)^. A more recent study conducted in Indonesia has found that the original eight-factor structure of the CEBQ showed adequate validity (based on Confirmatory Factor Analysis (CFA)), and stated that it is culturally appropriate for mothers of preschool children living in low- and middle-income countries^(11)^.

Socio-cultural and other related contexts play a role in shaping eating behaviours^(1)^. Understanding the local context is, thus, imperative to explaining the dynamics of the child feeding process^(1)^. The fast-progression of urbanisation and rapid socioeconomic and lifestyle changes in cities of developing countries are disrupting both access to food and feeding practices^(16)^. However, there is a limited evidence about child feeding and eating behaviours in most African countries such as Ethiopia. A recent study conducted in Addis Ababa found that the overall mean score for the eight scales of the CEBQ was close to the midpoint (2⋅5); however, emotional overeating (mean: 1⋅31, sd: 0⋅59) and slowness in eating (mean: 3⋅47, sd: 0⋅86) mark the opposite extremes of the spectrum^(17)^. The study also found a strong association between the eating behaviour scales and caregiver's controlling feeding practices^(17)^. Moreover, studies done among adolescents in Addis Ababa have also revealed that there is a cultural influence on perceptions of ‘healthy’ appearance and the presence of unhealthy weight control behaviour practices^(18,19)^. This indicates the importance of more research on eating behaviours that manifest in early childhood as they can be a precursor to maladaptive eating later in life. Targeting children's eating behaviours is important to prevent health-related problems, such as poor growth and obesity^(20,21)^. Developing countries including the study area are currently facing a double burden of malnutrition^(22)^. A recent study documented the co-existence among preschoolers in our study area, where a high prevalence of overweight/obesity (11⋅4 %) and undernutrition (19⋅6 %) was reported^(23)^. Therefore, having an instrument with the capacity to measure children's eating behaviours with good reliability and validity is crucial for early detection and monitoring of children at risk and to provide evidence to enhance theoretical development^(6)^. Validity of the CEBQ, however, remains under-researched in Sub-Saharan African countries. The present study, thus, aims to investigate the psychometric validity of the Ethiopian version of CEBQ in preschool children.

## Materials and methods

### Study design and procedures

We conducted a school-based cross-sectional study among 542 caregivers of children aged between three and six attending preschools in Addis Ababa, the capital city of Ethiopia. We used a multi-stage sampling technique to obtain a representative sample of study participants. The study examined all preschool children attending selected schools in Addis Ababa during the academic year 2018/19 and their parents/caregivers. The schools were selected by first stratifying the sub-cities into three strata by using socioeconomic status indicators. And finally, a simple random procedure was used to select students using school registers from each grade.

Parents/caregivers of the randomly selected students were recruited by sending an invitation to participate in the study through the school's teachers and the students’ communication books. Those willing to participate came to the schools at the appointed time. Informed consent was obtained after explaining the main purpose of the study. Finally, the parents/caregivers completed an interview-based questionnaire. The children's anthropometric measurements were taken the next day. We conducted a 7-h session to train interviewers on how to administer the CEBQ self-report interviews for the caregivers. The training session gave overviews of standard interview practices and the content of the CEBQ items both in English and in Amharic. In addition, the training also covered the use of certain interview probes and their purposes. There was also a demonstration of an interview using role-playing methods by the trainees.

### Ethical declaration

This study was conducted according to the guidelines laid down in the Declaration of Helsinki, and all procedures involving research study participants were approved by the ethical review board of Addis Ababa University under project number 0011. Written informed consent was obtained from all caregivers.

### Measures

#### Children's eating behaviour

The CEBQ is a retrospective, parental/caregiver reported questionnaire that has been used worldwide to examine eating behaviours of children aged 2–9 years^(9)^. The original version of CEBQ consists of 35 items that evaluates eight subscales of eating behaviour: food responsiveness (FR = 5 items), enjoyment of food (EF = 4 items), emotional overeating (EOE = 4 items), desire to drink (DD = 3 items), slowness in eating (SE = 4 items), satiety responsiveness (SR = 5 items), food fussiness (FF = 6 items) and emotional undereating (EUE = 4 items). The items are rated on a five-point Likert scale (1 = Never, 2 = Rarely, 3 = Sometimes, 4 = Often, 5 = Always).

#### Assessment of face validity

CEBQ was first translated by a qualified bilingual researcher whose mother tongue is Amharic into the Amharic version and then back translated into English by a different bilingual researcher whose mother tongue is Amharic to ensure the quality of the translation and maintain consistency in the questionnaire. The researchers were independent of the study to ensure the accuracy of the Amharic version. However, we faced some errors/discrepancies during the translation process due to the usage of wrong words, phrases being too complex and some were performed through the word to word translation which distorted the overall meaning of the questions, for 7 items of the questionnaire. Then, a consensual translation of the questionnaire was performed. Face validity was conducted for the 35 items of the CEBQ^(9)^. Two focus group discussions with a total of twenty parents, ten parents per each focus group which had comparable socio-demographic status with the study sample, were conducted with the aim of clarifying the wording of the instruments and their intended meaning. Some of the discussion points were concerned with how best to structure the items in a way that could be understood in the context of the country without losing the original meaning of the questions. Participants were also asked for ideas about improving the wording of questions that were perceived as vague or unclear; constructive feedback from parents at this point was used to refine the questionnaire. During the focus group discussions, nine of the thirty-five questions had a discrepancy between the translated questionnaire and their intended meaning. Several items seemed redundant using the equivalent Amharic terminologies: item 2 ‘My child eats more when worried’, item 9 ‘My child eats less when angry’, item 13 ‘My child eats more when annoyed’ and item 15 ‘My child eats more when anxious’. In addition, based on the constructive feedback from five parents, we decided to clarify and rephrase item 27 ‘My child eats more when s/he has nothing else to do’, item 30 ‘My child cannot eat a meal if s/he has had a snack just before’ and item 34 ‘If given the chance, my child would always have food in his/her mouth’. All of the items were then modified and rephrased based on the feedback received from the parents into more widely available and contextually acceptable phrases. The remaining items were retained in their original form since the parents understood the intended meaning of the items and there was no confusion with their meaning.

### Anthropometric measurements

Anthropometric measurements were taken to compute the children's BMI to assess the convergent validity of the CEBQ eight-factor tool. Weight was measured to the nearest 0⋅1 kg using an electronic portable scale (Seca), while height was measured in the standing position to the nearest 0⋅1 cm using a portable locally made stadiometer. We conducted an anthropometric measurement (weight and height) standardisation exercise among selected children and calculated the intra- and inter-observer technical errors of measurement (TEM). The intra-observer technical error of measurement for height was found to be 0⋅19 and for weight 0⋅12. The inter-observer technical error of measurement for height was found to be 0⋅21 and for weight 0⋅21. The coefficient of reliability was 97⋅5 %. All the measurements were found to be within an acceptable range.

### Demographic and socioeconomic characteristics

The children's sex and age (in completed years) were measured. The caregivers’ education levels were measured using a scale of (1) no formal education, (2) primary education, (3) secondary education, (4) technical school and above. Socioeconomic status was assessed through ownership of household assets and housing condition-related variables.

### Data management and analysis

We used the Stata version 15.0 statistical software package for data cleaning and analysis. Descriptive data were presented using frequency, percentage and mean (sd). All the analyses performed were pre-specified and based on the study objective.

The World Health Organization's (WHO) 2007 growth reference was used as a standard reference for classifying preschool children's BMI using the WHO's Anthro Plus software, version 1.0.21. Children's weight status was classified using the WHO 2007 growth reference for BMI-for-age cut-offs. Children that were 5 years and above with BAZ < −3 were classified as severely underweight, and children between −3 and −2 as underweight, between −2 and +1 as normal, between +1 and +2 as overweight and >+2 as obese^(24)^. Children aged below 5 years with BAZ < −3 were classified as severely underweight, and children between −3 and −2 as underweight, between −2 and +1 as normal, between +1 and +2 as at risk of overweight, between +2 and +3 as overweight and >+3 as obese^(25)^.

The socioeconomic status of the study households was determined using the PCA. Household asset ownership and variables related to housing conditions were used in the analysis to categorise households into wealth quintiles, ranging from poorest to the wealthiest.

The construct validity was assessed by subjecting the items of the instrument to CFA. The CFA was done to confirm whether the items were a good measure of the latent constructs, to check the item load on the proposed constructs and to consider the appropriateness of the factor structure^(26)^. CFA is considered to be the ‘gold standard’ for validating hypothesised factor structures (models) and provides a more accurate examination of the structure of the data and variable correlations^(27)^. First, Kaiser-Meyer-Olkin (KMO) test was done to indicate the adequacy of the sample for factor analysis; it was considered adequate if above 0⋅5. Moreover, for each CFA, we allowed intercorrelations between each of the factors and did not allow cross-factor loadings. We decided not to use *χ*^2^ statistics to evaluate model fit due to its sensitivity to sample size. Therefore, three common model fit indices were adopted to evaluate fit of the overall model: Comparative Fit Index (CFI), Tucker–Lewis Index (TLI) and root-mean-square error of approximation (RMSEA). Commonly used cut-off values were used in the present study: TLI/CFI > 0⋅95 (good fit), 0⋅90–0⋅95 (borderline fit) and <0⋅90 (poor fit); RMSEA <0⋅06 (good fit), 0⋅06–0⋅08 (fair fit), 0⋅08–0⋅10 (borderline fit) and >0⋅10 (poor fit)^(28)^. In addition, we judged the acceptability of model fit indices relative to the ideal values and re-specified the models based on standard regression coefficient loadings, modification indices and important theoretical grounds.

Bivariate correlations between each of the eight CEBQ subscales and the child's BMI *z*-scores were computed to assess convergent validity. Furthermore, discriminant validity was tested using factor–factor correlations. Cronbach's *α*s were also calculated to assess the internal reliability of the subscales.

## Result

### Participants characteristics

From a total of 542 caregivers we approached, 525 participated in the study, resulting in a response rate of 96⋅8 %. A total of 17 caregiver–child dyads were not included in the study because the caregivers did not have adequate time/refused to be interviewed. The socio-demographic characteristics of caregiver–child dyads are presented in Table 1. Most of the respondents (92⋅2 %) were the mothers of the index children. The mean age of the children was 4⋅5 years (±sd = 0⋅04). We found that 76⋅9 % (404) of the children had normal BMI-for-age scores. However, 8⋅0 % (42) children were overweight, and 2⋅9 % (15) were obese. Our findings also showed that 5⋅9 % (31) children were underweight (see Table 1).

**Table 1.** Characteristics of the preschool children and their caregivers in Addis Ababa, Ethiopia

Variables	*N*	%	Mean (sd)
Child's age (in year)			4⋅5 (0⋅04)
Child's sex			
Male	247	47	
Female	278	53	
Caregivers’ relation to the child			
Mother	484	92⋅2	
Father	14	2⋅7	
Nanny	10	1⋅9	
Others	17	3⋅2	
Caregivers’ educational status			
No formal education	59	11⋅2	
Primary education	131	24⋅9	
Secondary education	183	34⋅8	
Technical school and above	152	28⋅9	
Wealth index			
Poorest	105	20⋅1	
Poor	104	19⋅9	
Medium	104	19⋅9	
Wealthy	104	19⋅9	
Wealthiest	104	19⋅9	
Child's BMI			
Thin	31	5⋅9	
Normal weight	404	76⋅9	
At risk of Overweight	33	6⋅3	
Overweight	42	8⋅0	
Obese	15	2⋅9	

### The eight-factor confirmatory factor analysis

The KMO of the tool was 0⋅83, which shows the data were suited for factor analysis. The analysis commenced with 35 items and involved a series of iterations, based on the accepted methods of identifying the best model. We tested the eight factors (35 items) of the original CEBQ. Of these, seven items were removed based on their low factor loading (<0⋅30): one item from food responsiveness ‘Even if my child is full up s/he finds room to eat his/her favorite food’, one item from desire to drink ‘My child is always asking for a drink’, two items from food fussiness ‘My child refuses new foods at first’ and ‘My child is difficult to please with meals’, one item from satiety responsiveness ‘My child cannot eat a meal if s/he has had a snack just before’, one item from slowness in eating ‘My child eats more and more slowly during the course of a meal’ and one item from emotional undereating ‘My child eats more when she is happy’. The finding that such items had a low factor loading is similar to other studies^(11,15)^. The fitness of the eight-factor model (28 items) after the removal of low loading items had RMSEA = 0⋅09 (90 % CI 0⋅087, 0⋅096); CFI = 0⋅70 and TLI = 0⋅64, providing a poor model fit (see Table 2).

**Table 2.** Confirmatory factor analysis of Children's Eating Behaviour Questionnaire, Addis Ababa, Ethiopia

Models	CFI	TLI	RMSEA	90 % CI for RMSEA
**Model^a^**	0⋅65	0⋅61	0⋅08	(0⋅079, 0⋅086)
**Model^b^**	0⋅70	0⋅64	0⋅09	(0⋅087, 0⋅096)
**Model^c^**	0⋅92	0⋅90	0⋅05	(0⋅045, 0⋅055)

CFI, Comparative Fit Index; TLI, Tucker–Lewis Index; RMSEA, root-mean-square error of approximation; CI, confidence interval.

a Model: Baseline model which is the original eight-factor model with 35 items.

b Model: the eight-factor model after the low loading items were removed (28 items).

c Model: the modified and final model fit after correlation of residuals and identification of some cross loadings items.

After reviewing the modification indices, however, correlation of residuals was conducted. We also identified some cross loadings that may have potentially contributed to the poor model fit. One of the cross loadings identified was a reverse-scored item from the Satiety Responsiveness subscale ‘My child has a big appetite’, which was also loaded on the Enjoyment of Food factor (in an opposite direction). This item was then specified to load only on Enjoyment of Food. The rationale for making this modification is that a child with a ‘big appetite’ may appear to enjoy food more and might describe how much s/he wants to eat^(9)^. This was similar to other studies^(29,30)^. The other cross loading was one of the items of the Emotional Overeating subscale ‘My child eats more when s/he has nothing else to do’, which had a significant cross loading on the Food Responsiveness factor so we loaded the item onto Food responsiveness. Based on the literature, the loading of this item onto Food Responsiveness is a clear reflection that this item describes external eating, a type of eating behaviour basic to food responsiveness^(9)^. This finding has also been found elsewhere^(10)^. After making these modifications, we found RMSEA = 0⋅05 (90 % CI 0⋅045, 0⋅055); CFI = 0⋅92 and TLI = 0⋅90, providing an acceptable model fit (see Table 2). The revised enjoyment of food and food responsiveness scales had five items each including those items that were previously loaded on another scale (see Table 3).

**Table 3.** Standardised regression weight for the modified eight-factor CEBQ tool in Addis Ababa, Ethiopia

Domain	Item	Name of the item	Estimate
Enjoyment of food	Item 1	My child loves food	0⋅768
	Item 5	My child is interested in food	0⋅844
	Item 16	My child looks forward to mealtimes	0⋅330
	Item 18	My child enjoys eating	0⋅577
	Item 3	My child has a big appetite	−0⋅811
Food responsiveness	Item 9	My child is always asking for food	0⋅505
	Item 11	If allowed to, my child would eat too much	0⋅470
	Item 15	Given the choice, my child would eat most of the time	0⋅421
	Item 30	If given the chance, my child would always have food in his/her mouth	0⋅518
	Item 23	My child eats more when s/he has nothing else to do	0⋅499
Emotional overeating	Item 2	My child eats more when worried	0⋅483
	Item 10	My child eats more when annoyed	0⋅759
	Item 12	My child eats more when anxious	0⋅816
Desire to drink	Item 25	If given the chance, my child would drink continuously throughout the day	0⋅742
	Item 27	If given the chance, my child would always be having a drink	0⋅856
Food fussiness	Item 7	My child enjoys tasting new foods	0⋅611
	Item 17	My child enjoys a wide variety of foods	0⋅694
	Item 18	My child is interested in tasting food s/he hasn't tasted before	0⋅679
Satiety responsiveness	Item 19	My child leaves food on his/her plate at the end of a meal	0⋅685
	Item 20	My child gets full before his/her meal is finished	0⋅572
	Item 21	My child gets full up easily	0⋅829
Slowness in eating	Item 22	My child finishes his/her meal quickly	0⋅559
	Item 23	My child eats slowly	0⋅549
	Item 24	My child takes more than 30 minutes to finish a meal	0⋅446
Emotional undereating	Item 25	My child eats less when angry	0⋅677
	Item 26	My child eats less when s/he is tired	0⋅624
	Item 27	My child eats less when upset	0⋅728

CEBQ, Children's Eating Behaviour Questionnaire.

### Discriminant and convergent validity

The results of discriminant validity were assessed using factor–factor correlations shown in Table 4. CEBQ subscales of food approach were positively correlated with each other, while food avoidance behaviours were correlated among themselves. In addition, a negative correlation was observed between categories except desire to drink. Overall, children's eating behaviours coefficients represented small to large relation based on Cohen's guidelines^(31)^. There was a highly significant correlation with satiety responsiveness and slowness in eating (*r* = 0⋅53, *P* < 0⋅01) and with enjoyment of food and food responsiveness (*r* = 0⋅49, *P* < 0⋅01). This indicates that children who are more responsive to food cues also tend to enjoy their food. Furthermore, enjoyment of food was moderately negatively related to food fussiness (*r* = 0⋅41, *P* < 0⋅01) and slowness in eating (*r* = 0⋅31, *P* < 0⋅01). This indicates that children tend to eat more slowly and are fussier in their eating tend to enjoy food less. Our finding shows that food responsiveness was positively correlated with emotional overeating (*r* = 0⋅45, *P* < 0⋅01) (see Table 4).

**Table 4.** Correlations between the CEBQ scales, child's BMI in Addis Ababa, Ethiopia

Pearson correlation coefficient								
Children's eating behaviour								
**Children's eating behaviour**	Enjoyment of food	Food responsiveness	Emotional overeating	Desire to drink	Food fussiness	Satiety responsiveness	Slowness in eating	Emotional undereating
**Enjoyment of food**	1⋅000							
**Food responsiveness**	0⋅499**	1⋅000						
**Emotional overeating**	0⋅239**	0⋅457**	1⋅000					
**Desire to drink**	0⋅069	0⋅126**	0⋅041	1⋅000				
**Food fussiness**	−0⋅418**	−0⋅363**	−0⋅066	−0⋅123**	1⋅000			
**Satiety responsiveness**	−0⋅227**	−0⋅063	−0⋅245**	0⋅134**	−0⋅053	1⋅000		
**Slowness in eating**	−0⋅315**	−0⋅171**	−0⋅285**	0⋅072	0⋅062	0⋅530**	1⋅000	
**Emotional undereating**	−0⋅108*	−0⋅022	−0⋅082*	0⋅071	−0⋅073	0⋅294**	0⋅351**	1⋅000
**Child's BMI-for-age**	0⋅057	0⋅054	0⋅166**	−0⋅054	0⋅113**	−0⋅135**	−0⋅124**	0⋅012

CEBQ, Children's Eating Behaviour Questionnaire, BMI, Body Mass Index.

*P < 0⋅05, **P < 0⋅01.

In addition, the result of convergent validity of the eight subscales with child nutritional status showed a significant positive correlation with emotional overeating scale (*r* = 0⋅16, *P* < 0⋅01) and food fussiness (*r* = 0⋅11, *P* < 0⋅01), while it was negatively correlated with satiety responsiveness (*r* = −0⋅13, *P* < 0⋅01) and slowness in eating scale (*r* = −0⋅12, *P* < 0⋅01). Meanwhile, enjoyment of food, food responsiveness, desire to drink and emotional undereating scales were not significantly correlated with child's BMI (see Table 4).

### Descriptive mean score and internal reliability of the scales

The internal reliability of the scales, measured with Cronbach's *α*, ranged from 0⋅50 to 0⋅79. All the scales scored higher than 0⋅70, except for food responsiveness and slowness in eating. Most of the subscales scored close to the midpoint (2⋅5). Emotional overeating (mean: 1⋅24, sd: 0⋅02) and food responsiveness (mean: 1⋅83, sd: 0⋅03) had the lowest scores, while satiety responsiveness (mean: 3⋅57, sd: 0⋅04) had the highest score (see Table 5).

**Table 5.** Children's Eating Behaviour Questionnaire descriptive statistics and internal reliability in Addis Ababa, Ethiopia

Subscales	Mean (sd)	Cronbach *α* (# of items)
**Enjoyment of food**	3⋅01 (0⋅03)	0⋅79 (5)
**Food responsiveness**	1⋅83 (0⋅03)	0⋅62 (5)
**Emotional overeating**	1⋅24 (0⋅02)	0⋅72 (3)
**Desire to drink**	2⋅96 (0⋅06)	0⋅77 (2)
**Food fussiness**	2⋅40 (0⋅46)	0⋅75 (3)
**Satiety responsiveness**	3⋅57 (0⋅04)	0⋅72 (3)
**Slowness in eating**	3⋅53 (0⋅04)	0⋅50 (3)
**Emotional undereating**	3⋅04 (0⋅05)	0⋅71 (3)

## Discussion

The present study was conducted with the aim of validating CEBQ among preschool children in Ethiopia. It also has assessed eating behaviour pattern with child's BMI among preschoolers in Ethiopia. We found that the CEBQ evidenced a reasonable fit to the data based on the CFA results, indicating that an eight-factor structure was the best solution for our sample corresponding to the original study in the UK^(9)^. The Ethiopian version of CEBQ has also shown a correlation with child's BMI scores and a good internal reliability except for food responsiveness and slowness in eating scales.

The CFA reveals that the eight-factor structure is suitable for our sample, which was similar to other studies conducted in developing countries such as Indonesia^(11)^ – the original eight-factor structure of the CEBQ showed adequate content validity and recommended that this too, can be used for all preschool children living in low- and middle-income countries. A study in Thailand^(32)^ also supported the cross-cultural utility of the CEBQ as a tool in the assessment of children. Studies from more developed countries such as Iceland^(33)^, the USA^(10)^ and Australia^(6)^ further support the use of the instrument as a measure of the original eight distinct eating style dimensions. Some studies conducted in non-European countries, however, have shown that the eight-factor CEBQ is not suitable. For instance, as noted earlier, a study done in China concluded that although the CEBQ was a valuable psychometric instrument, it may be affected by cultural differences and proposed a seven-factor solution, with factor ‘food responsiveness’ (FR) split into two^(15)^. Furthermore, two cohort studies from Singapore also revealed a revised six-factor structure and a revised seven-factor structure of the CEBQ were more appropriate for examining appetitive traits in children aged 3–6 years^(29,30)^. The reason for the observed difference might be due to the type of analysis used to determine the validity as most of the studies conducted in different parts of the world have used explanatory factor analysis/PCA^(13,15,34)^. Another study, however, has implied that CFA is considered to be a more accurate examination of the structure of the data and variable correlations than PCA^(27)^. Furthermore, establishing the number of discrete constructs a scale measures are of fundamental importance given that combining possibly discrete subscales into one decreases the sensitivity of the CEBQ which has implications for its usefulness in detecting problematic eating behaviours and measuring treatment gains^(33)^. The other reason might be related to the sample size. Any factor analysis needs an adequate sample size and some studies in non-European countries have relatively small samples, which might jeopardise the accuracy of the results of the factor structure.

This version of CEBQ differed from the original eight-factor model mainly on some item factor loadings. For instance, item 3 ‘My child has a big appetite’ was loaded on the enjoyment of food scale. Even though this item is typically considered a satiety responsive item, on the original tool, we found that it was best loaded on the enjoyment of food scale. The rationale for this was that besides having a relatively better loading coefficient, is that caregivers interpreted this item as child being easy to feed and, therefore, enjoying their food^(30)^. This issue is similar to other studies, where this item was merged with the enjoyment of food scale^(29,30)^. In addition, item 23 ‘My child eats more when s/he has nothing else to do’ was loaded on food responsiveness when it originally belonged to the emotional overeating scale. This was in line with another study from the USA where this item was placed on the food responsiveness scale^(10)^. Other studies have also commonly reported items from the emotional overeating subscale combined with the items from the food responsiveness subscale^(13,30)^. This might be due to the interpretation of the item since caregivers, considered eating when bored to be different from emotional eating behaviours^(35)^ and mostly relate this item to child's food responsive behaviour of overeating^(12,30)^. It may also be related to caregiver's inability to assess if their children ate more when feeling certain emotions as evidenced by the low mean score reported in this study.

The other pertinent finding is our convergent validity test, revealing that the emotional overeating scale is the only food approach behaviour that has a significant positive relation to child's BMI score. This is in line with studies conducted in Portugal^(36)^ and Chile^(34)^ where emotional overeaters tend to eat more during negative emotional states^(9)^ and are more prone to become overweight. However, some studies have found no association between emotional overeating and BMI score^(13,29)^, suggesting that eating triggered by emotional stress may not be accurately perceived by parents in children at younger ages, and associations with weight might only emerge at later time points^(29)^.

The other interesting finding is that satiety responsiveness and slowness in eating scale are significantly related to a lower child's BMI score. This is similar to findings from Singapore^(29,30)^, Portugal^(36)^ and Australian^(6)^ studies. This shows that children that are satiety responsive and that tend to eat more slowly have a lower weight status. This indicates that eating behaviours contribute to poor nutritional status among children particularly in our study area. Indeed, a recent study conducted in Addis Ababa revealed a high prevalence of stunting (19⋅6 %) and wasting (3⋅2 %) among preschool children^(23)^. Some studies, however, have found no association between child's BMI scores and any of the scales^(13,15)^. Many developing countries such as Ethiopia are currently facing a double burden of malnutrition. Thus, it is essential to include examples of healthy eating behaviours in national nutritional programmes to improve the nutritional status of children.

The factor-to-factor correlation test showed that none of the correlations is high enough (*r* ≥ 0⋅8) to show any overlap of factors, thereby disclaiming the existence of construct redundancy and supporting the theoretical link between constructs in CEBQ^(33)^. CEBQ subscales that belong to the two categories of food approach and food avoidance indicate a significant positive correlation with those in the same category and a negative correlation with those with a different category except the desire to drink. This is similar to findings from Portugal^(36)^ and Sweden^(13)^. Our reliability test found that all the scales have a good reliability score, except for food responsiveness and slowness in eating scales. The reason for the low *α* might be because Cronbach *α* is quite sensitive to the number of items in the scale which have been seen in other similar studies^(15,34)^. It is common to find scales with quite low Cronbach values when the items are less than 10^(37)^. Future studies should further check the applicability and sensitivity to the cultural difference of the above scales.

The present study has significant strengths, including (1) an adequate sample size with the use of probability sampling techniques and (2) the use of standardisation protocols for anthropometric measurements, which help obtain accurate and precise anthropometric measurements and reduces errors. However, there are limitations to the present study that should be considered. First, the study's cross-sectional nature hinders the inference of causal conclusions between children's eating behaviour and their BMI score as it was only assessed on one occasion. Second, social desirability bias cannot be ruled out since the measurements were based on the caregivers’ self-reporting rather than direct observation of children's eating behaviour. Additionally, caregivers may respond differently in an interview, *v.* self-administered anonymous questionnaire. Further replication of the study among low-income countries is essential to improve our concept of eating behaviours.

## Conclusion

The present study validates the CEBQ among sub-Saharan African countries, specifically Ethiopia. The CEBQ evidenced a reasonable fit to the data based on the CFA results which support the eight-factor structure in a sample of caregivers reporting behaviours of preschool-aged children. The Ethiopian version of CEBQ scales has also demonstrated convergent validity as expected with children's BMI *z*-scores and a good internal reliability, except for food responsiveness and slowness in eating scales.

## References

[1] Savage JS, Fisher JO & Birch LL (2007) Parental influence on eating behavior: conception to adolescence. J Law Med Ethics 35, 22–34. 17341215 10.1111/j.1748-720X.2007.00111.x PMC2531152

[2] Lewinsohn PM, Holm-Denoma JM, Gau JM, (2005) Problematic eating and feeding behaviors of 36-month-old children. Int J Eat Disord 38, 208–219. 16211627 10.1002/eat.20175 PMC1351337

[3] Marcus MD & Kalarchian MA (2003) Binge eating in children and adolescents. Int J Eat Disord 34, S47–S57. 12900986 10.1002/eat.10205

[4] Linscheid TR, Budd KS & Rasnake LK (2003) Pediatric feeding problems. In Handbook of pediatric psychology, pp. 481–498 [M. C. Roberts Ed.]. New York: The Guilford Press.

[5] Bryant-Waugh R, Markham L, Kreipe RE, (2010) Feeding and eating disorders in childhood. Int J Eat Disord 43, 98–111. 20063374 10.1002/eat.20795

[6] Mallan KM, Liu W-H, Mehta RJ, (2013) Maternal report of young children's eating styles. Validation of the Children's Eating Behaviour Questionnaire in three ethnically diverse Australian samples. Appetite 64, 48–55. 23333562 10.1016/j.appet.2013.01.003

[7] Van Strien T, Frijters JE, Bergers GP, (1986) The Dutch Eating Behavior Questionnaire (DEBQ) for assessment of restrained, emotional, and external eating behavior. Int J Eat Disord 5, 295–315.

[8] Archer LA, Rosenbaum PL & Streiner DL (1991) The children's eating behavior inventory: reliability and validity results. J Pediatr Psychol 16, 629–642. 1744810 10.1093/jpepsy/16.5.629

[9] Wardle J, Guthrie CA, Sanderson S, (2001) Development of the Children's Eating Behaviour Questionnaire. J Child Psychol Psychiatry 42, 963–970. 11693591 10.1111/1469-7610.00792

[10] Domoff SE, Miller AL, Kaciroti N, (2015) Validation of the Children's Eating Behaviour Questionnaire in a low-income preschool-aged sample in the United States. Appetite 95, 415–420. 26247701 10.1016/j.appet.2015.08.002 PMC4589500

[11] Purwaningrum D, Arcot J, Hadi H, (2020) A cultural adaptation and validation of a child eating behaviour measure in a low- and middle-income country. Public Health Nutr 23, 1–8. 32383413 10.1017/S136898001900510X PMC10200370

[12] Sleddens EF, Kremers SP & Thijs C (2008) The Children's Eating Behaviour Questionnaire: factorial validity and association with Body Mass Index in Dutch children aged 6–7. Int J Behav Nutr Phys Act 5, 1–9. 18937832 10.1186/1479-5868-5-49 PMC2612017

[13] Svensson V, Lundborg L, Cao Y, (2011) Obesity related eating behaviour patterns in Swedish preschool children and association with age, gender, relative weight and parental weight-factorial validation of the Children's Eating Behaviour Questionnaire. Int J Behav Nutr Phys Act 8, 134. 22152012 10.1186/1479-5868-8-134 PMC3286377

[14] Falciglia GA, Couch SC, Gribble LS, (2000) Food neophobia in childhood affects dietary variety. J Am Diet Assoc 100, 1474–1481. 11138439 10.1016/S0002-8223(00)00412-0

[15] Cao Y-T, Svensson V, Marcus C, (2012) Eating behaviour patterns in Chinese children aged 12–18 months and association with relative weight-factorial validation of the Children's Eating Behaviour Questionnaire. Int J Behav Nutr Phys Act 9, 5. 22272572 10.1186/1479-5868-9-5 PMC3311563

[16] Berhane HY, Ekström E-C, Jirström M, (2018) What influences urban mothers’ decisions on what to feed their children aged under five—the case of Addis Ababa, Ethiopia. Nutrients 10, 1142. 10.3390/nu10091142 PMC6164347 30135354

[17] Gebru NW, Gebreyesus SH, Yirgu R, (2021) The relationship between caregivers’ feeding practices and children's eating behaviours among preschool children in Ethiopia. Appetite 157, 104992. 33049339 10.1016/j.appet.2020.104992

[18] Yirga B, Gelaw YA, Derso T, (2016) Disordered eating attitude and associated factors among high school adolescents aged 12–19 years in Addis Ababa, Ethiopia: a cross-sectional study. BMC Res Notes 9, 1–7. 27927224 10.1186/s13104-016-2318-6 PMC5143448

[19] Tuffa TA, Gebreyesus SH, Endris BS, (2020) Unhealthy weight control behaviors among Ethiopian female adolescents. Int J Eat Disord 53, 525–532. 31944363 10.1002/eat.23227

[20] Jansen PW, Roza SJ, Jaddoe VW, (2012) Children's eating behavior, feeding practices of parents and weight problems in early childhood: results from the population-based Generation R Study. Int J Behav Nutr Phys Act 9, 130. 23110748 10.1186/1479-5868-9-130 PMC3543222

[21] Kwon KM, Shim JE, Kang M, (2017) Association between picky eating behaviors and nutritional status in early childhood: performance of a picky eating behavior questionnaire. Nutrients 9, 463. 10.3390/nu9050463 PMC5452193 28481251

[22] Abdullah A (2015) The double burden of undernutrition and overnutrition in developing countries: an update. Curr Obes Rep 4, 337–349. 26627492 10.1007/s13679-015-0170-y

[23] Berhane HY, Jirström M, Abdelmenan S, (2020) Social stratification, diet diversity and malnutrition among preschoolers: a survey of Addis Ababa, Ethiopia. Nutrients 12, 712. 10.3390/nu12030712 PMC7146462 32156006

[24] Van den Broeck J, Willie D & Younger N (2009) The World Health Organization child growth standards: expected implications for clinical and epidemiological research. Eur J Pediatr 168, 247–251. 18670787 10.1007/s00431-008-0796-9

[25] WHO (2006) WHO child growth standards: length/height-for-age, weight-for-age, weight-for-length, weight-for-height and body mass index-for-age: methods and development. Geneva, Switzerland: World Health Organization.

[26] Steiger JH (1990) Structural model evaluation and modification: an interval estimation approach. Multivariate Behav Res 25, 173–180. 26794479 10.1207/s15327906mbr2502_4

[27] Kim H-J (2008) Common factor analysis versus principal component analysis: choice for symptom cluster research. Asian Nurs Res (Korean Soc Nurs Sci) 2, 17–24. 25031108 10.1016/S1976-1317(08)60025-0

[28] Lt H & Bentler PM (1999) Cutoff criteria for fit indexes in covariance structure analysis: conventional criteria versus new alternatives. Struct Equ Modeling 6, 1–55.

[29] Quah PL, Fries LR, Chan MJ, (2019) Validation of the Children's Eating Behavior Questionnaire in 5 and 6 year-old children: the GUSTO Cohort Study. Front Psychol 10, 824. 31031683 10.3389/fpsyg.2019.00824 PMC6470280

[30] Quah PL, Cheung YB, Pang WW, (2017) Validation of the Children's Eating Behavior Questionnaire in 3 year old children of a multi-ethnic Asian population: the GUSTO cohort study. Appetite 113, 100–105. 28232104 10.1016/j.appet.2017.02.024 PMC5384631

[31] Cohen J (2013) Statistical power analysis for the behavioral sciences. New York: Routledge.

[32] Sirirassamee T & Hunchangsith P (2016) Children's eating behavior questionnaire: factorial validation and differences in sex and educational level in Thai school-age children. Southeast Asian J Trop Med Public Health 47, 1325–1334. 29634198

[33] Njardvik U, Klar EK & Thorsdottir F (2018) The factor structure of the Children's Eating Behaviour Questionnaire: a comparison of four models using confirmatory factor analysis. Health Sci Rep 1, e28. 30623064 10.1002/hsr2.28 PMC6266355

[34] Santos JL, Ho-Urriola JA, González A, (2011) Association between eating behavior scores and obesity in Chilean children. Nutr J 10, 108. 21985269 10.1186/1475-2891-10-108 PMC3213088

[35] Hayman Jr. LW, Lee HJ, Miller AL, (2014) Low-income women's conceptualizations of emotional- and stress-eating. Appetite 83, 269–276. 25218718 10.1016/j.appet.2014.09.005 PMC4253654

[36] Viana V, Sinde S & Saxton J (2008) Children's Eating Behaviour Questionnaire: associations with BMI in Portuguese children. Br J Nutr 100, 445–450. 18275626 10.1017/S0007114508894391

[37] Pallant J (2001) Checking the reliability of a scale. SPSS Survival Manual, version. Open University Press. ISBN: 9781000248722.

